# A 16-miRNA Prognostic Model to Predict Overall Survival in Neuroblastoma

**DOI:** 10.3389/fgene.2022.827842

**Published:** 2022-06-29

**Authors:** Jiepin Wang, Dong Xiao, Junxiang Wang

**Affiliations:** ^1^ Shenzhen Children’s Hospital, Shenzhen, China; ^2^ Shantou University Medical College, Shantou, China

**Keywords:** neuroblastoma, miRNA, prognostic model, overall survival, bioinformatic analysis

## Abstract

Neuroblastoma is the most malignant childhood tumor. The outcome of neuroblastoma is hard to predict due to the limitation of prognostic markers. In our study, we constructed a 16-miRNA prognostic model to predict the overall survival of neuroblastoma patients for early diagnosis. A total of 205 DE miRNAs were screened using RNA sequencing data from GSE121513. Lasso Cox regression analysis generated a 16-miRNA signature consisting of hsa-let-7c, hsa-miR-135a, hsa-miR-137, hsa-miR-146a, hsa-miR-149, hsa-miR-15a, hsa-miR-195, hsa-miR-197, hsa-miR-200c, hsa-miR-204, hsa-miR-302a, hsa-miR-331, hsa-miR-345, hsa-miR-383, hsa-miR-93, and hsa-miR-9star. The concordance index of multivariate Cox regression analysis was 0.9, and the area under the curve (AUC) values of 3-year and 5-year survival were 0.92 and 0.943, respectively. The mechanism was further investigated using the TCGA and GSE90689 datasets. Two miRNA–gene interaction networks were constructed among DEGs from two datasets. Functional analysis revealed that immune-related processes were involved in the initiation and metastasis of neuroblastoma. CIBERSORT and survival analysis suggested that lower CD8 T-cell proportion and higher SPTA1 expressions were related to a better prognosis. Our study demonstrated that the miRNA signature may be useful in prognosis prediction and management improvement.

## Introduction

Neuroblastoma is one of the most common childhood tumors that accounts for nearly 28% of pediatric cancer diagnosed in Europe and America. The 5-year event-free survival rate varies from 37% to 69% among different races. The prevalence of neuroblastoma is higher in Blacks and Native Americans (Heck et al., 2009; Henderson et al., 2011). However, the underlying etiology is not clear currently. Possible causes include maternal medication use, childhood infections, and others (Cook, 2004; Menegaux, 2004). The adrenal gland is the most common primary site resulting in hypertension. The patient can be asymptomatic or present with abdominal pain, swollen belly, and constipation as the number and location of neuroblastoma vary (Vo et al., 2014). According to the International Neuroblastoma Risk Group (INRG) staging system, patients can be divided into groups of low, intermediate, and high risk. Tumor stages, age at diagnosis, histology category, tumor differentiation, and MYCN status are the major factors of INRG classification schema (Monclair et al., 2009). Furthermore, neuroblastoma is also contributed by genetic factors. However, there is a lack of sensitive and specific biomarkers, which cause approximately 50% of patients to have distant metastasis at diagnosis, especially in a high-risk group (DuBois et al., 1999). Therefore, it is necessary to understand the mechanism of neuroblastoma initiation and metastasis and identify the prognostic predictors of neuroblastoma.

miRNA is a group of small single-stranded non-coding RNA molecules of about 22 nucleotides (Bartel, 2018). In tumorigenesis, miRNA can function as a tumor suppressor gene or oncogene. It has been reported that miRNAs exert multiple functional roles in the development of neuroblastoma. The high expression of miR-380-5p was highly correlated with the poor prognosis of neuroblastoma with MYCN amplification by repressing p53 (Swarbrick et al., 2010). By targeting MYCN and AKT2, miR-184 had a positive effect on inhibiting neuroblastoma cell survival (Foley et al., 2010). As a tumor suppressor, miR-497 regulated WEE1 in neuroblastoma cells (Creevey et al., 2013). However, few studies have focused on the miRNA prognostic model of neuroblastoma.

The bioinformatic analysis of miRNA sequences has become popular in general cancer research recently. A large number of online databases and tools have contributed a lot to understanding and predicting tumor development. The Gene Expression Omnibus (GEO) database and The Cancer Genome Atlas (TCGA) database freely distribute high-throughput molecular abundance data. These databases provide access to publicly available data for researchers. Lasso Cox regression is widely used in prognostic prediction for different kinds of tumors (Luo et al., 2017; Zhang et al., 2019). In our study, we constructed a miRNA prognostic model *via* integrated bioinformatic analysis. Our study was designed first to screen the differentially expressed miRNAs in neuroblastoma tissue samples to normal tissue samples. Among the DE miRNAs, we established a 16-miRNA prognostic model that included hsa-let-7c, hsa-miR-135a, hsa-miR-137, hsa-miR-146a, hsa-miR-149, hsa-miR-15a, hsa-miR-195, hsa-miR-197, hsa-miR-200c, hsa-miR-204, hsa-miR-302a, hsa-miR-331, hsa-miR-345, hsa-miR-383, hsa-miR-93, and hsa-miR-9star based on the lasso Cox regression analysis. A predictive nomogram was generated and internally validated by time-dependent receive operating characteristic (ROC) and calibration curves. Furthermore, we performed the Kaplan–Meier survival analysis of each miRNA in the prognostic model. In order to investigate the role of prognostic miRNAs in neuroblastoma initiation and metastasis, we established two miRNA–gene interaction networks between prognostic miRNAs and DEGs from the TCGA dataset and GSE90689 dataset. Gene Ontology (GO) analysis on the DAVID database was performed based on the DEGs from the miRNA–gene interacting pairs. Finally, we performed CIBERSORT analysis to investigate tumor-infiltrating immune cells. Our study might be helpful in exploring the role of miRNAs in neuroblastoma development and identifying candidate prognostic biomarkers which can be the potential molecular targets of therapeutic drugs.

## Materials and Methods

### Data Collection and Processing

**FIGURE 1:**
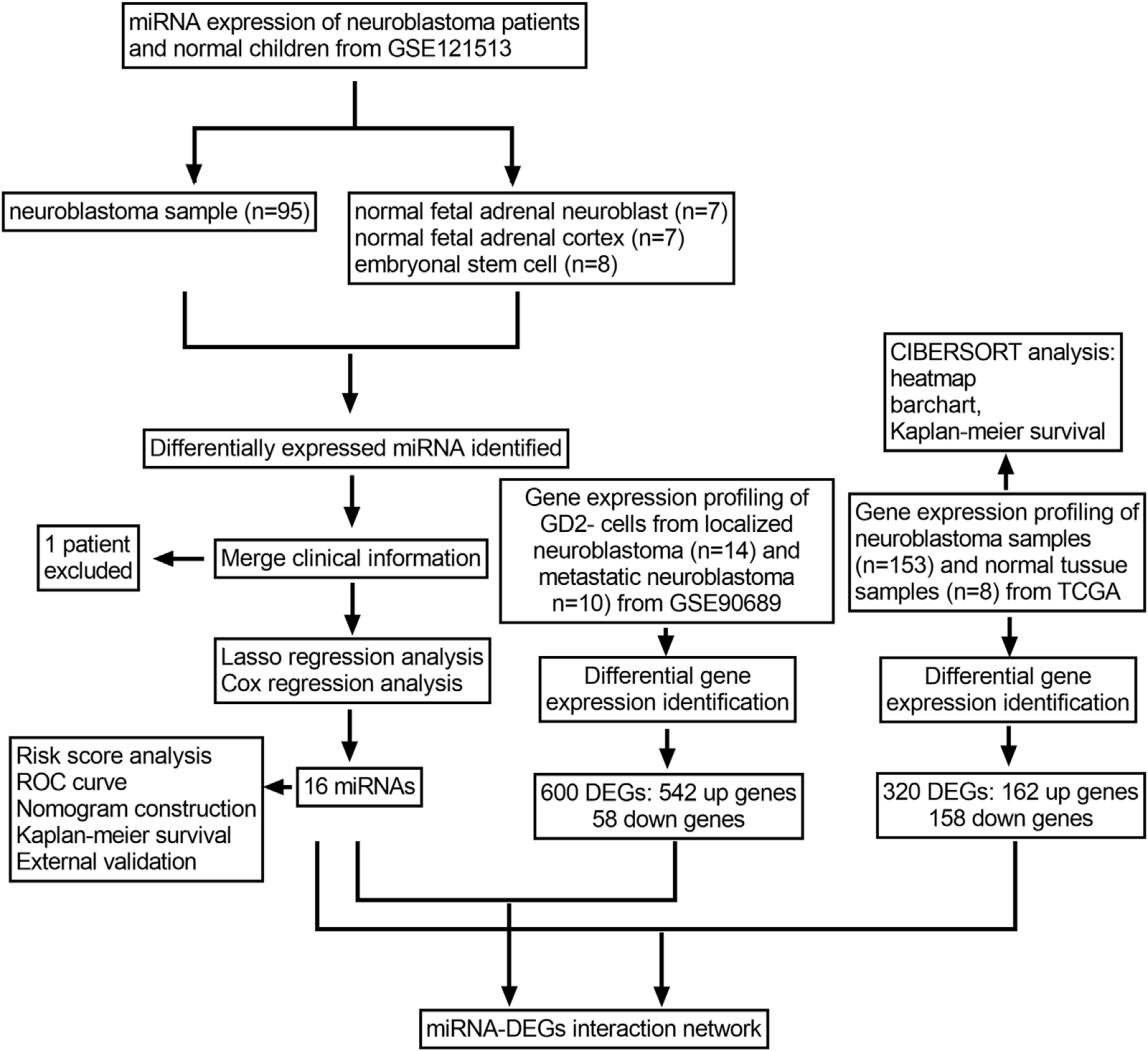
Flowchart of the whole research process.

The whole analysis process is presented in (Figure 1). First, the miRNA expression profile of GSE121513 was downloaded from the Gene Expression Omnibus (GEO) database (http://www.ncbi.nlm.gov/geo). GSE121513 consisted of 95 neuroblastoma samples, seven normal fetal adrenal neuroblasts, seven normal fetal adrenal cortices, and eight embryonic stem cells. GSE121513 was based on GPL25696 platforms (430 miRNA panel_AppliedBiosystems). The transcriptome expressions of 153 neuroblastoma samples and eight normal tissue samples were obtained from The Cancer Genome Atlas (TCGA) database (http://tcga-data.nci.nih.gov/tcga). Furthermore, GSE90689 was downloaded, which consisted of 13 bone marrow cells from healthy donors, 20 bone marrow cells from localized neuroblastoma, 14 GD2 cells from localized neuroblastoma, and 10 GD2 cells from metastatic neuroblastoma. For further investigation, we chose 24 samples of GD2 negative cells from localized or metastatic neuroblastoma. GSE90689 was based on GPL6480 (Agilent-014850 Whole Genome Microarray 4 × 44K G4112F). All the data have open access and required no additional approval from an ethics committee.

### Differentially Expressed miRNAs and Gene Identification

The limma package (version: 3.460) installed in R was used to identify DE miRNAs in three separate comparisons: neuroblastoma samples versus the normal fetal adrenal neuroblast, neuroblastoma samples versus the normal fetal adrenal cortex, and neuroblastoma samples versus embryonic stem cells. In each comparison group, the differentially expressed miRNAs were defined with a *p*-value < 0.05 and |log2FC|>2. DE miRNAs of three groups were combined for further analysis. The limma package was also used to screen the DEGs. In TCGA database, the transcriptome expression levels were analyzed between the neuroblastoma samples and normal tissue samples. In order to investigate the metastatic process of neuroblastoma, the DEGs between the localized and metastatic neuroblastoma in GSE90689 were screened. The DEGs were defined with a *p*-value < 0.5 and |log2FC|>mean (abs(logFC)) + 2*sd (abs(logFC)). The DEGs in two datasets would be further analyzed and integrated with the DE miRNAs.

### miRNA-Related Prognostic Model Establishment

The glmnet package and survival package installed in R were used to construct the Lasso Cox model. First, we calculated the correlation expression of each DE miRNA with the survival information of patients using a univariate Cox model. The DE miRNAs of with a *p*-value < 0.05 were considered of statistical significance. Second, we used the least absolute shrinkage and selection operator (Lasso) regression analysis to screen the statistically significant DE miRNAs in the univariate Cox model. The range of optimal lambda value was identified *via* cross-validation. Third, we selected the DE miRNAs derived from Lasso regression to construct the prognostic model. Based on the prognostic model, we performed the multivariate Cox regression analysis to test the concordance index. A forest plot was drawn using the survmier package. Finally, we used the survival package and Rms package to establish a nomogram of 3-year survival prediction and 5-year survival prediction and calibrations of the prognostic model.

### ROC Curve Establishment and Survival Analysis

In order to test the prognostic performance of the miRNA prognostic model, a time-dependent receive operating characteristic (ROC) curve was generated by comparing the sensitivity and specificity. Furthermore, the Kaplan–Meier survival curves of the prognostic model and each miRNA were generated using the survival package. Patients were divided into high- and low-risk groups, according to the median risk score. The status and survival time of patients for each miRNA were compared between the aforementioned two groups.

### Outer Validation of the miRNAs in the Prognostic Model

The miRNA expression profile of GSE86889 was downloaded from the Gene Expression Omnibus (GEO) database (http://www.ncbi.nlm.gov/geo). GSE86889 consisted of 15 NB cell lines and 20 neuroblastoma samples. GSE86889 was based on GPL14943 platforms (Agilent-029297 Human miRNA Microarray). In order to investigate the roles of the prognostic model in neuroblastoma, the expressed levels of 16 miRNAs were compared between NB cell lines and neuroblastoma.

### miRNA–Gene Network Construction and Functional Analysis

miRwalk (http://mirwalk.uni-hd.de/) is an online miRNA database containing the experimentally validated miRNA-target interaction pairs (Dweep et al., 2014). The prognostic miRNAs were uploaded into miRwalk to screen the miRNA–gene targets. We downloaded and merged the miRNA–gene pairs with two groups of DEGs described earlier. The DEGs that interacted with miRNAs were screened. Then, the miRNA–DEG pairs of two groups were identified and downloaded by Cytoscape software (version: 3.7.2) to generate miRNA–gene networks. We used DAVID (http://david.ncifcrf.gov) to perform the GO analysis. Sub-analysis included biological processes (BP), cellular components (CC), and molecular function (MF) based on the Gene Ontology (GO) database. We considered that *p*-value<0.05 was statistically significant. DEGs interacting with miRNA were uploaded into the DAVID database to investigate the role of miRNAs in the progression and metastasis of neuroblastoma.

### CIBERSORT Analysis

To investigate the underlying mechanism of neuroblastoma development, we performed the CIBERSORT analysis to estimate the proportions of immune cells of neuroblastoma in TCGA dataset. From the gene expression profiles, CIBERSORT was an effective method to predict the fraction of specific cells (Newman et al., 2015). In our study, 22 kinds of immune cells were selected as a reference. The proportions of immune cells were calculated by the expression levels of gene neuroblastoma tissues. The subsequent Kaplan–Meier analysis of 22 kinds of immune cells was performed. The interacting genes from the TCGA dataset enriched in the biological processes of the immune response were also determined by Kaplan–Meier analysis.

## Results

### Differentially Expressed miRNA Identification

**FIGURE 2:**
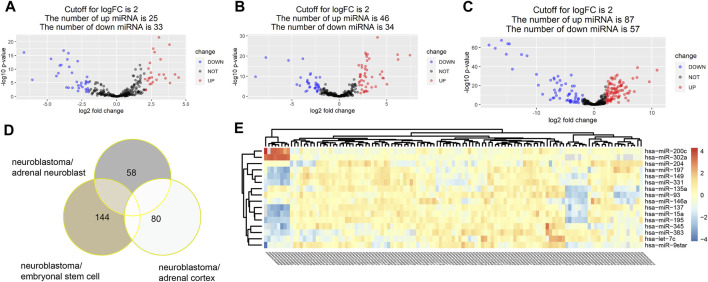
With the threshold at *p* value<0.05 and |log2FC|>2, 58,144 and 80 DE miRNAs were identified in turn in three groups of comparison. A total of 25 upregulated miRNAs and 33 downregulated miRNAs were identified in the group of neuroblastoma versus adrenal neuroblast **(A)**. Also, 46 upregulated miRNAs and 34 downregulated miRNAs were identified in the group of neuroblastoma versus adrenal cortex **(B)**, and 87 upregulated miRNAs and 57 downregulated miRNAs were identified in the group of neuroblastoma versus embryonal stem cells **(C)**. Totally, 205 DE miRNAs were identified **(D,E)**.

With the threshold at *p*-value < 0.05 and |log2FC|>2, 58,144 and 80 DE miRNAs were identified in turn in three groups of comparison. A total of 25 upregulated miRNAs and 33 downregulated miRNAs were identified in the group of neuroblastoma versus adrenal neuroblast (Figure 2A). Also, 46 upregulated miRNAs and 34 downregulated miRNAs were identified in the group of neuroblastoma versus adrenal cortex (Figure 2B), and 87 upregulated miRNAs and 57 downregulated miRNAs were identified in the group of neuroblastoma versus embryonal stem cells (Figure 2C). Totally, 205 DE miRNAs were identified (Figure 2D,E).

### Construction of the miRNA Signature

**FIGURE 3:**
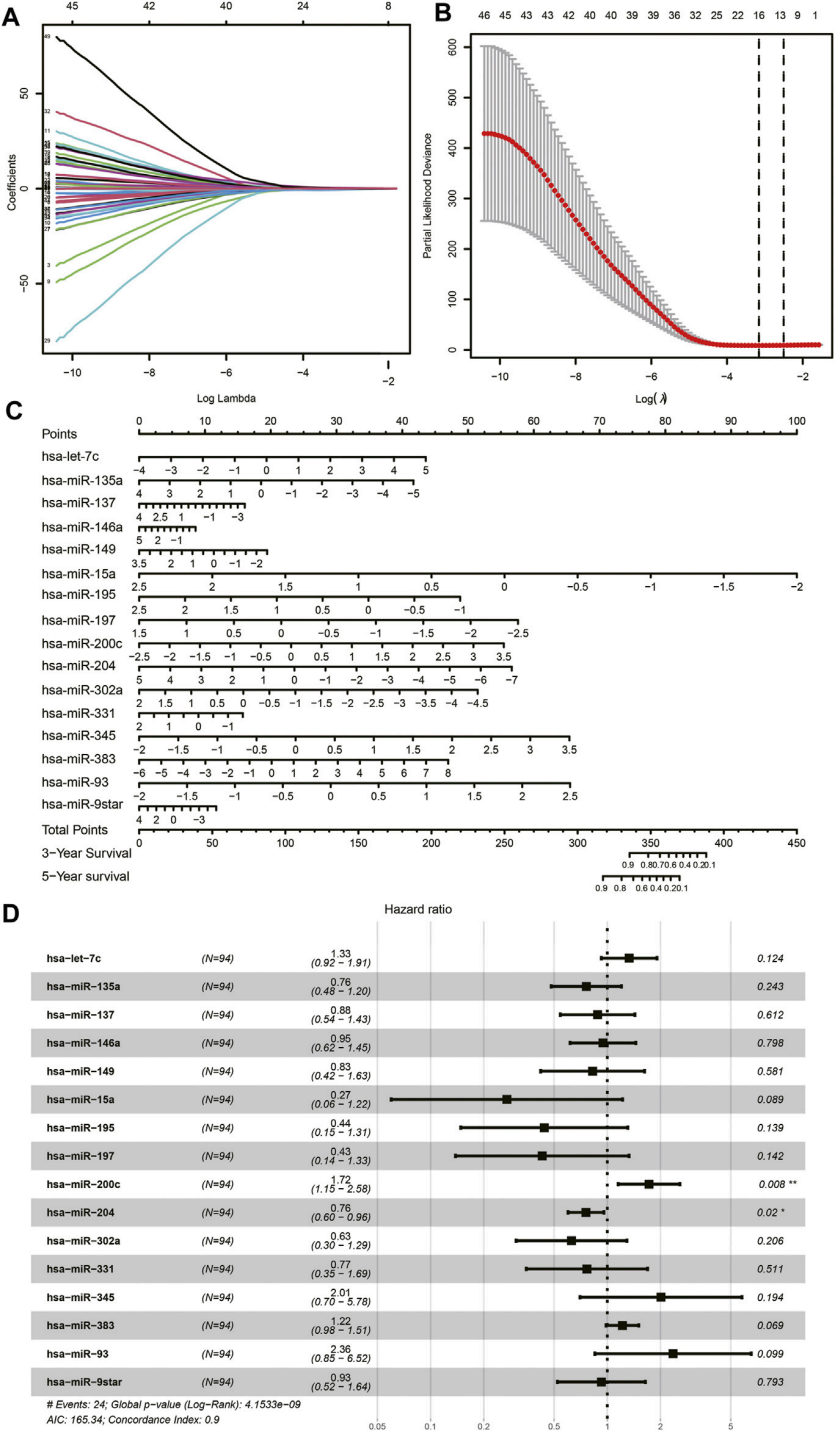
Lasso Cox regression analysis. **(A)** Lasso coefficient profiles of the miRNAs. **(B)** Cross-validation to tune parameter selection in Lasso regression analysis. **(C)** Nomogram. **(D)** Forest plot of multivariate Cox regression.

Among the total of 205 DE miRNAs, univariate Cox regression analysis showed 64 miRNAs had a significant association with the survival information of patients including the status and survival time. Among these miRNAs, Lasso regression analysis was performed for further filtration. The regression coefficient diagrams were present including Lasso coefficient profiles and cross-validation parameters. Coefficient profiles decreased as the lambda value increased (Figure 3A, B). In total, 16 prognostic miRNAs were retained including hsa-let-7c, hsa-miR-135a, hsa-miR-137, hsa-miR-146a, hsa-miR-149, hsa-miR-15a, hsa-miR-195, hsa-miR-197, hsa-miR-200c, hsa-miR-204, hsa-miR-302a, hsa-miR-331, hsa-miR-345, hsa-miR-383, hsa-miR-93, and hsa-miR-9star. The aforementioned 16 miRNAs were further subjected to multivariate Cox regression analysis to verify whether they were independent prognostic miRNAs for neuroblastoma. A forest map of the correlation between each miRNA and survival was generated after the multivariate Cox analysis (Figure 3D). The value of the concordance index was 0.9. The hazard ratios of hsa-miR-200c and hsa-miR-204 were statistically significant. We considered that the prognostic model of neuroblastoma patients consisted of 16 miRNAs. These 16 miRNAs were incorporated to develop the nomogram for predicting 3 and 5 years of survival probability. As shown in the nomogram, hsa-miR-15a made the largest contribution to the prognosis, followed by hsa-miR-93 and hsa-miR-345 (Figure 3C).

### Validation of the Prognostic Model

**FIGURE 4:**
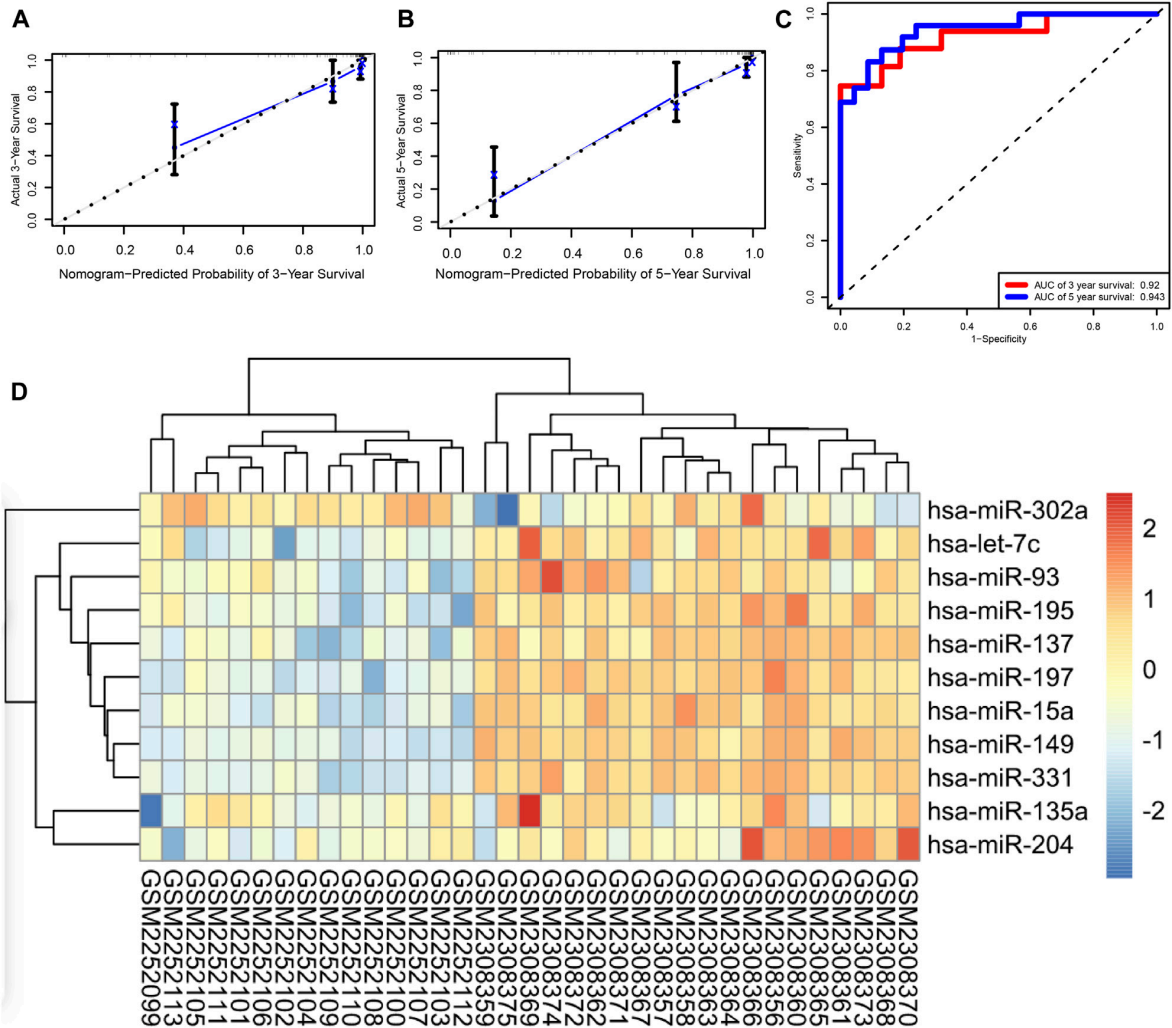
**(A,B)** Calibration curves of the nomogram, **(C)** ROC curve, and **(D)** external validation.

Based on the expression levels of 16 miRNAs, a risk score was calculated for each patient. Patients were divided into high- and low-risk groups, and each group had 47 patients. Furthermore, we demonstrated the ROC and the calibration curves to examine the predictive accuracy of the prognostic models. The predicted probability of 3 and 5 years of survival by the nomogram is nearly consistent with the actual survival (Figures 4A,B). The ROC curve showed that AUCs of 3 and 5 years of survival were 0.92 and 0.943, respectively (Figure 4C). In order to verify the role of miRNAs in neuroblastoma, we performed an external validation based on GSE86889. A total of 11 miRNAs in the prognostic model including hsa-miR-302a, hsa-let-7c, hsa-miR-93, hsa-miR-195, hsa-miR-195, hsa-miR-137, hsa-miR-197, hsa-miR-15a, hsa-miR-149, hsa-miR-331, hsa-miR-135a, and hsa-miR-204 were differentially expressed between NB cell lines and neuroblastoma samples (Figure 4D).

### Survival Analysis of miRNAs

**FIGURE 5:**
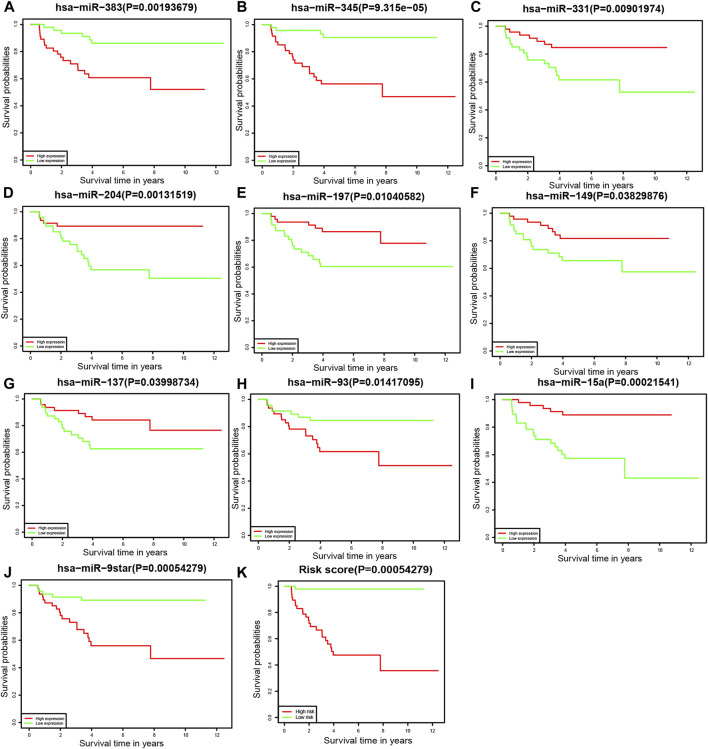
Kaplan–Meier survival analysis **(A)** hsa-miR-383, **(B)** hsa-miR-345, **(C)** hsa-miR-331, **(D)** hsa-miR-204, **(E)** hsa-miR-197, **(F)** hsa-miR-149, **(G)** hsa-miR-137, **(H)** hsa-miR-93, **(I)** hsa-miR-15a, **(J)** hsa-miR-9star, and **(K)** the prognostic model.

To further understand the relationship between each miRNA and the overall survival rate, we performed the Kaplan–Meier survival analysis among 94 patients. Of the prognostic model, the expression levels of 10 miRNAs were statistically significant to the survival status. The overall survival rates of neuroblastoma patients with higher hsa-miR-383, hsa-miR-345, hsa-miR-93, and hsa-miR-9star expression levels were lower. By contrast, patients with higher hsa-miR-331, hsa-miR-204, hsa-miR-197, hsa-miR-149, hsa-miR-137, and hsa-miR-15a expression levels had a higher overall survival rate of neuroblastoma (Figure 5).

### Differentially Expressed Gene Identification

**FIGURE 6:**
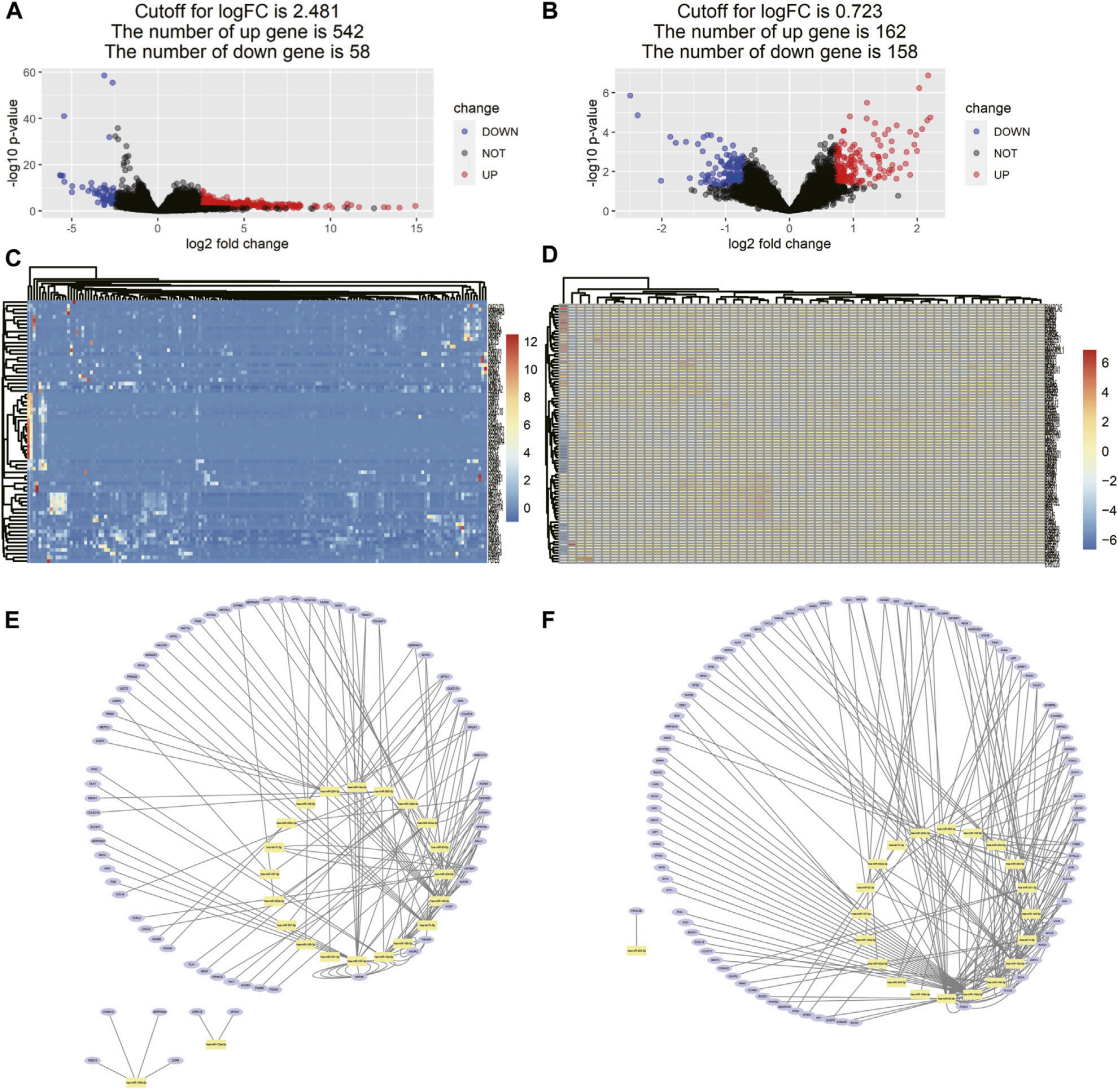
Differentially expressed gene identification and miRNA–DEG interaction network. **(A)** Volcano map of DEMs between neuroblastoma samples and normal tissue samples from TCGA. **(B)** Volcano map of DEMs between GD2 cells from localized neuroblastoma and metastatic neuroblastoma from GSE90689, **(C,D)** heatmaps of DEMs interacting with 16 miRNA in two data series, and **(E,F)** miRNA–gene interaction networks in two data series. The yellow dots represent each miRNA in the prognostic model. The blue dots represent DEGs interacting with miRNA in the prognostic model in two data series.

As the cutoff for logFC was 2.481, there were 600 DEGs including 542 upregulated and 58 downregulated genes screened in 161 tissue samples of TCGA dataset (Figures 6A,C). As for the comparison between GD2 cells of localized and metastatic neuroblastoma of GSE90689, 320 DEGs consisting of 162 upregulated genes and 158 downregulated genes were identified with the threshold at *p* < 0.05 and |log2FC|>0.723 (Figures 6B,D).

### Constructed miRNA–Gene Interaction Network and Functional Analysis of Interacting DEGs

**FIGURE 7:**
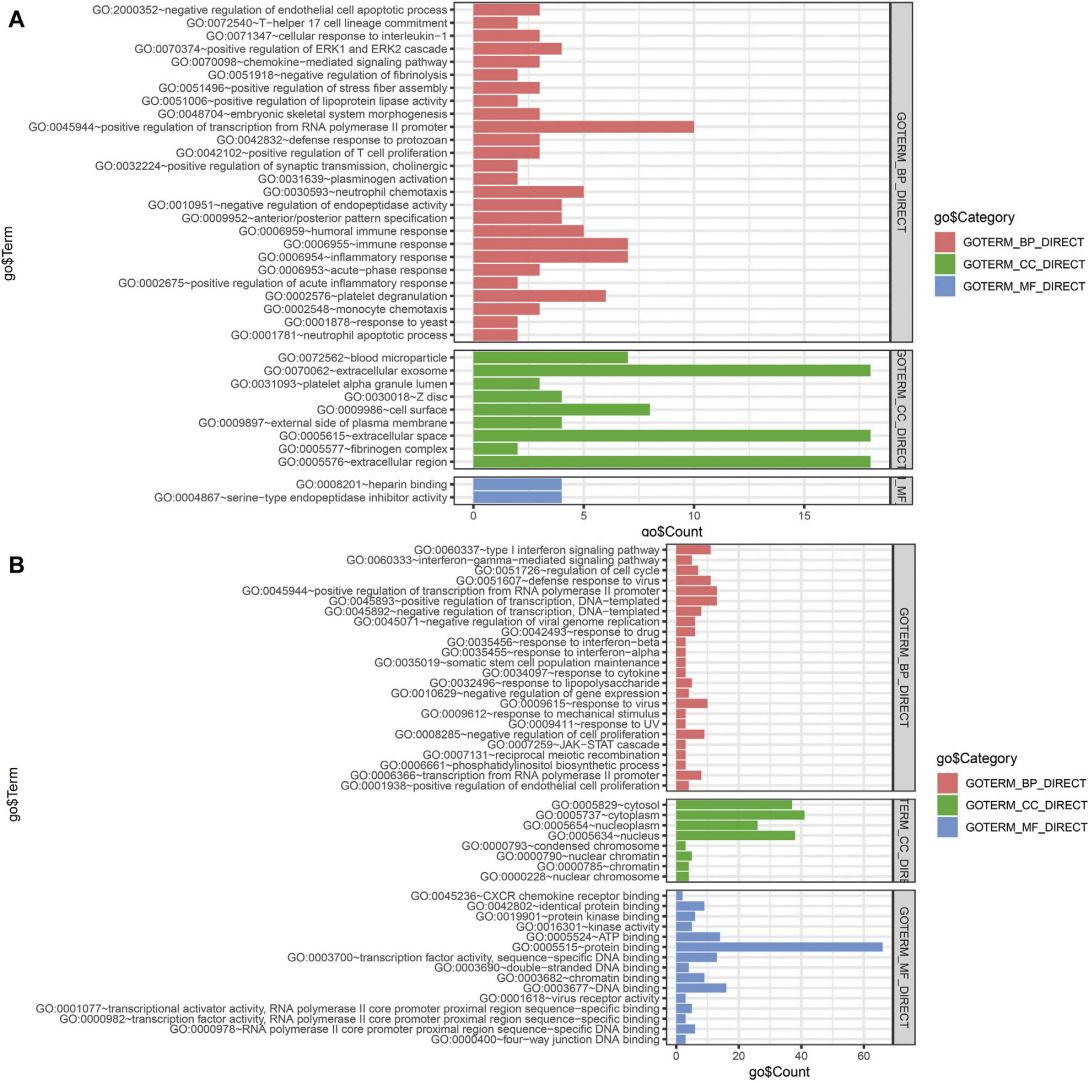
Functional enrichment analysis of DEGs interacting with prognostic miRNAs. **(A)** GO analysis of DEGs of tissue samples from TCGA database. **(B)** GO analysis of DEGs of tissue samples from GSE90689. BP, biological process; CC, cellular component; MF, molecular function; DEGs, differentially expressed genes; GO, Gene Ontology.

The miRNA–gene interaction table was downloaded using the miRwalk database. Two miRNA–gene interaction networks were constructed by Cytoscape software (Figures 6E,F). In TCGA database analysis, 71 DEGs interacted with 15 miRNAs in the prognostic model. GO-based BP analysis showed that interacting genes were majorly enriched in the positive regulation of transcription from RNA polymerase II promoter (GO: 0045944), immune response (GO: 0006955), inflammatory response (GO: 0006954), and platelet degranulation (GO: 0002576). These interacting genes were majorly located in the extracellular exosome (GO: 0070062) and were functional in heparin binding (GO: 0008201) and serine-type endopeptidase inhibitor activity (GO: 0004867). In the GSE90689 analysis, 93 DEGs interacted with 15 miRNAs in the prognostic model. GO-based BP analysis showed that interacting genes were majorly enriched in the type I interferon signaling pathway (GO: 0060337) and positive regulation of transcription. (GO: 0045944 and GO: 0045893). Most of the interacting genes were located in the cytosol (GO: 0005829), cytoplasm (GO: 0005737), and nucleus (GO: 0005634). These genes were functional in protein binding (GO: 0005515), DNA binding (GO: 0003677), and ATP binding (GO: 0005524). All GO terms whose *p*-value < 0.05 are presented in (Figure 7).

### Tumor Infiltration Immune Cell Model

**FIGURE 8:**
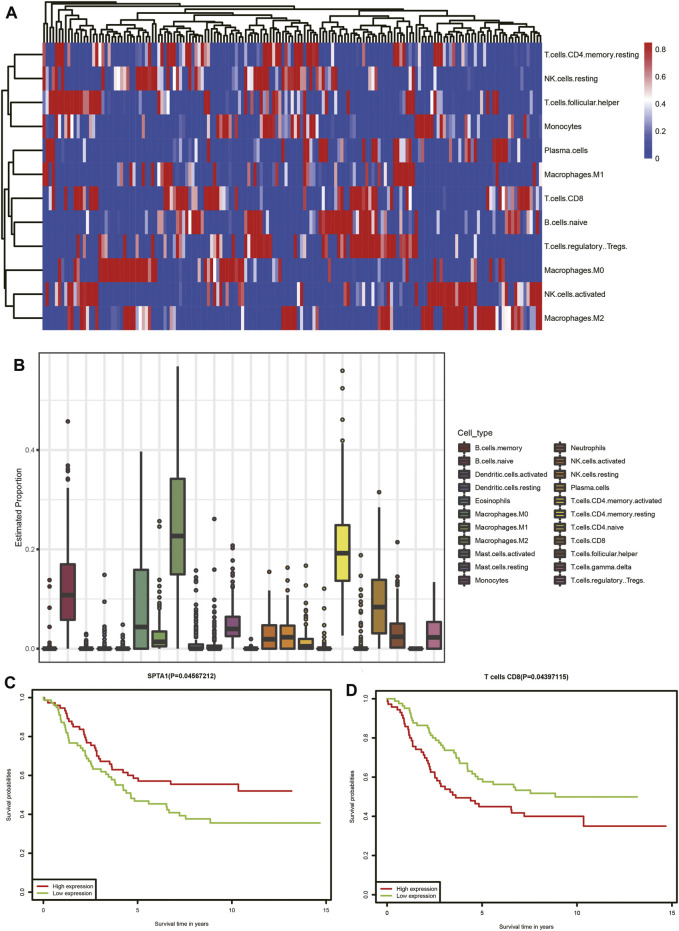
Tumor-infiltrating immune cell analysis of TCGA dataset. **(A)** Heatmap of immune cells. Each column represents a neuroblastoma tissue sample. Each row represents one type of immune cells. Colors from blue to red represent increasing proportions of immune cells in samples. **(B)** Proportions of 22 types of immune cells. **(C)** Association of the SPTA1 expression level and overall survival in TCGA neuroblastoma dataset. **(D)** Association of CD8 T-cell infiltration and overall survival in TCGA neuroblastoma dataset.

In the functional analysis of interacting genes in TCGA dataset, most of the genes were enriched in biological processes associated with the immune response. We performed CIBERSORT analysis to investigate the proportions of immune cells. The heatmap and bar chart of proportions of immune cells are presented in (Figures 8A,B). According to the result, we identified that neuroblastoma tissue consisted of relatively high percentages of naive B cells, M0 and M2 macrophages, resting memory CD4 T cells, and CD8 T cells. The subsequent survival analysis of immune cells indicated that patients with a high proportion of CD8 T cells had lower survival rates (Figure 8D). Furthermore, we selected 11 interacting genes including *CCL16*, *CCR9*, *CHST4*, *CXCL3*. *IFNG*, *IL6*, *MS4A1*, *POU2AF1*, *SPTA1*, *TNFSF9*, and *TREM1* from the humoral immune response (GO: 0006959), immune response (GO: 0006955), and positive regulation of T-cell proliferation (GO: 0042102)Among 11 interacting genes associated with the immune response, Kaplan–Meier analysis indicated the high expression of SPTA1 was related to a better prognosis of neuroblastoma patients (Figure 8C).

## Discussion

Neuroblastoma is the most malignant tumor of early childhood and a significant cause of childhood death. Patients with neuroblastoma, especially those in the high-risk group, have a poor prognosis. Therefore, it is necessary to understand the molecular biology of neuroblastoma which can provide new tools for clinicians in diagnosis and treatment. It has been widely reported that miRNAs have a positive/negative effect on the progression of neuroblastoma. However, the number of therapeutic/prognostic markers is still limited. In order to reduce mortality and improve prognosis, it is necessary to establish a model of prognostic prediction which might be useful for guiding the clinical treatments of neuroblastoma patients for early diagnosis.

In our study, we investigated the differentially expressed miRNAs between neuroblastoma and normal tissue including the fetal adrenal neuroblast, fetal adrenal cortex, and embryonic stem cells from RNA-seq data from GSE121513. Among 205 DE miRNAs, additional analyses including univariate, Lasso, and multivariate Cox regression analyses were performed to construct a prognostic model. The prognostic model consisted of hsa-let-7c, hsa-miR-135a, hsa-miR-137, hsa-miR-146a, hsa-miR-149, hsa-miR-15a, hsa-miR-195, hsa-miR-197, hsa-miR-200c, hsa-miR-204, hsa-miR-302a, hsa-miR-331, hsa-miR-345, hsa-miR-383, hsa-miR-93, and hsa-miR-9star. The concordance index of forest plot was 0.9. The AUCs of the ROC curve for predicting 3 and 5 years of survival of the prognostic model were 0.92 and 0.943, respectively. Based on the miRNAs from the prognostic model, Kaplan–Meier survival analysis was performed to investigate the correlation between each miRNAs and overall survival. The high expression levels of hsa-miR-383, hsa-miR-345, hsa-miR-93, and hsa-miR-9star were relevant to a poor prognosis in neuroblastoma patients. By contrast, the high expression levels of hsa-miR-331, hsa-miR-204, hsa-miR-197, hsa-miR-149, hsa-miR-137, and hsa-miR-15a were relevant to a good prognosis. Finally, we constructed a miRNA signature containing 16 miRNAs as a prognostic model for neuroblastoma patients.

Among the miRNAs in the prognostic model, most of them were reported to be associated with tumor initiation and progression. However, the functions of most miRNAs in neuroblastoma development remain largely undefined. Ooi et al. (2018) identified miR-204 as a tumor suppressor miRNA, which could be repressed by MYCN and associated with the poor outcome in neuroblastoma patients. Additionally, the PHOX2B gene as a common mutation in neuroblastoma cases was downregulated by miR-204 in neuroblastoma cells (Bachetti et al., 2015). Chava et al. (2020) found that miR-15a binds to MYCN mRNA to inhibit the expression of MYCN. Furthermore, upregulation of miR-15a can induce CXCR4 inhibition, which downregulated neuroblastoma growth (Klein et al., 2018). Apoptosis of neuroblastoma was induced by the overexpression of miR-149 targeting CDC43, BCL2, Akt1, and E2F1 (Lin et al., 2010; Mao et al., 2019). Apoptosis can also be induced by miR-146a, which repressed the expression of BCL11A (Li et al., 2018). Althoff et al. (2013) found that KDM1A knockdown can repress neuroblastoma cell viability, and KDM1A was validated as a target of miR-137. They considered miR-137 as a tumor suppressor in neuroblastoma. The tumor-suppressing effects of miR-204, miR-15a, miR-149, and miR-137 were consistent with those of our Kaplan–Meier survival analysis. The roles of miRNAs were also reported in other kinds of tumors of the nervous system. He et al. (2013) found that downregulation of miR-383 can induce the invasion of glioma cells by activating AKT signaling and targeting insulin-like growth factor 1 receptor (He et al., 2013). Song et al. (2016) suggested that miR-204 suppressed the proliferation, migration, and invasion of glioblastoma cells by inhibiting ATF2.

To investigate the role of prognostic miRNAs in the initiation and metastasis of neuroblastoma, we constructed two miRNA–gene interaction networks among two groups of DEGs. The predicted target genes for each prognostic miRNA were put into the DAVID database to perform GO-based enrichment analysis. Most of the predicted target genes from the TCGA database were enriched in the immune response including the regulation of inflammatory cells and chemokines, which suggested that miRNAs might be the possible regulators in the immune microenvironment during neuroblastoma initiation. In order to confirm the role of the immune microenvironment in neuroblastoma progression, we performed the CIBERSORT analysis to investigate the tumor-infiltrating immune cells. We found the proportions of naïve B cells, M0 and M2 macrophages, resting memory CD4 T cells, and CD8 T cells were high. The proportion of CD8 T cells was found to be significantly associated with overall survival. CD8 T cells, also known as cytotoxic T cells can recognize specific antigens produced by cancer cells (Al-Shura, 2020). The T-cell–mediated response can be evoked in neuroblastoma development by different specific antigens including MAGE, GAGE, and NY-ESO-1 (Rodolfo et al., 2003). In our study, CD8 T cells were negatively related to the overall survival, which means that the antigens recognized by CD8 T cells in neuroblastoma had a negative effect on the prognosis of neuroblastoma patients. We also performed the survival analysis of interacting genes in TCGA dataset and found that SPTA1 was positively related to the overall survival. SPTA1 encoded a family of molecular scaffold proteins including 22 spectrin repeats, which determine the shape of cells. Downregulation of alphaII-spectrin can induce a frequent break during neuron cell migration (Trinh-Trang-Tan et al., 2014). Furlan et al. found that 80% of chromaffin cells in the adrenal medulla were originated from the migrating SCPs. Chromaffin cells were identified as the source of neuroblastoma (Furlan et al., 2017; Tsubota and Kadomatsu, 2018). We assumed that the downregulation of SPTA1 decreased the expression of spectrin, which affect the normal migration of neural crest cells to chromaffin cells. Matsushita et al. (2012) also found that tumor cells lacking highly antigenic mutant spectrin-beta2 can be promoted *via* T-cell dependent selection. However, there were few studies focused on SPTA1 as the potential therapeutic molecular target in neuroblastoma. It was necessary to further explore the potential of SPTA1 in immune therapy.

To investigate the association between the prognostic model and metastasis, we performed the functional analysis of interacting DEGs between GD2 cells between localized and metastatic neuroblastoma in GSE90689. The enrichment analysis showed that most of the interacting DEGs significantly involved in biological processes were related to the interferon signaling pathway. Studies have shown that the activation of the interferon pathway can alter the growth and metastasis of different cancers (Izawa et al., 2002; Snijders et al., 2014; Singh et al., 2020). However, there is another opinion that type I interferon was produced by malignant cells and can elicit immune responses *via* tumor cell in intrinsic or extrinsic ways (Musella et al., 2017). Based on the results, we considered that the evaluation of the IFN signature expression can be a potential prognostic marker for patients with newly diagnosed neuroblastoma.

In conclusion, our study identified a 16-miRNA prognostic model predicting the overall survival of neuroblastoma patients. The nomogram of a prognostic model can be a reliable tool in the selection of personalized treatment at early diagnosis. The utility was tested with high AUC values. Furthermore, our study indicated that CD8 T cells and SPTA1 might be important in neuroblastoma initiation, while type I interferons played a significant role in neuroblastoma metastasis. However, further studies are needed to elucidate the function and mechanism of all these biomarkers in neuroblastoma progression.

## Data Availability

The original contributions presented in the study are included in the article/supplementary material; further inquiries can be directed to the corresponding author.
